# Food and Taxation

**Published:** 1903-10-24

**Authors:** 


### Foods and Taxation.

The proposal of Mr. Chamberlain to impose a light taxation upon the chief articles of food which we import both from foreign countries and from our own colonies, in order that by a remission of the tax in the latter case we may unite more closely than at present the bonds between the different portions of the Empire, and that, by the power of remitting it in the former, we may gain a means of negotiating with our chief commercial rivals as to the admission of our own manufactures into their respective territories, is one that is likely to engage the attention of the public for a long period, and that may possibly lead to unexpected developments in more than one direction. Whether the proposal be sound or unsound, whether it would produce the good effects anticipated by its author, or the evils predicted by his opponents, it would at present be premature to pronounce; but it is at least the proposal of a statesman, habituated to take wide views of great questions, and able to do justice to their greatness. The speeches in which it has been advocated have been brilliant displays of this habit and of this ability, and have presented a marked contrast to those of the mere politicians, whose vapid formulas and worn-out shibboleths have been suggestive of the archers of the fourteenth century, drawn out in battle array against magazine rifles and modern artillery. But the passive resistance offered to change by a dead weight of ignorance and stupidity is so considerable as to render it rash to predict a speedy victory for the more powerful and better-equipped assailant; and it is quite possible that some such mere begging of the question as is displayed by a parrot cry for "free food" may be sufficient to sway the votes of large numbers of the electors to whom, in the last issue, this great imperial question must be submitted. The case is one in which the scattered members of the medical profession may render great and real service to the country. There may, of course, be arguments against Mr. Chamberlain's policy, although, so far, they can hardly be said to have been produced; but if they should hereafter be brought forward, educated men in country districts could do nothing more patriotic than to examine into their validity, and to try to enlighten their neighbours with regard to them. If we are to retain a policy of unrestricted free imports, let our population at least be assisted to understand the consequences and to count the cost.

If the final result of the education of the people upon this question should be a decision to tax certain articles of food, not merely for the purpose of obtaining revenue, but also for other reasons, an important question might arise as to the possibility of using such taxation to guide and control demand. That it can be applied for this purpose has been shown by many examples. Almost precisely two hundred years ago Lord Methuen concluded a treaty by which the wines of Portugal were admitted into this country at duties less by one-third than those placed upon the wines of France; and the result was a speedy change in the popular taste, by which Port came almost to supersede the previously universal Burgundy. At the present time we hear of much alarm as to a supposed tendency to physical degeneration among our people; and there can be little doubt, if such a tendency exist, that it may be due, or at all events that it may to some extent be promoted, by changes for the worse in the staple character of the national food. That this is the case in Scotland has been very positively asserted; and the partial abandonment of the former oatmea diet of the peasantry has been assigned as a probable cause of changes for the worse in their capabilities. Dr. Johnson, it will be remembered, defined "oats" as "food for horses in England and for men in Scotland," and his definition occasioned the pertinent inquiry, "where are there such men, or such horses?" Now there is only too much reason to believe that many forms of food, which have become popular among the poor, mainly because little or no care or skill is required in order to place them upon the table, or because, in proportion to weight or bulk, they are capable of being regarded as "cheap," or like Sam Weller's crumpets, as "filling at the price," have been by no means blameless with regard to any amount of the assumed degeneration which may be genuine. If the principle of the taxation of food be once admitted, as, sooner or later, it seems sure to be, there will be no subject better worth inquiry than how the dietary of the mass of the people is composed, and to what extent it might be improved by such modifications of the tariff as to favour the better materials and to check the admission of the worse.

